# Decreased serum phosphate levels are a useful biomarker to predict occurrence and severity of cytokine release syndrome in chimeric antigen receptor T‐cell therapy

**DOI:** 10.1111/bjh.18504

**Published:** 2022-10-11

**Authors:** Naokazu Nakamura, Yasuyuki Arai, Toshio Kitawaki, Tomoyasu Jo, Chisaki Mizumoto, Junya Kanda, Momoko Nishikori, Kouhei Yamashita, Akifumi Takaori‐Kondo

**Affiliations:** ^1^ Department of Hematology and Oncology Graduate School of Medicine, Kyoto University Kyoto Japan; ^2^ Department of Clinical Laboratory Medicine Graduate School of Medicine, Kyoto University Kyoto Japan; ^3^ Center for Research and Application of Cellular Therapy Kyoto University Hospital Kyoto Japan

**Keywords:** chimeric antigen receptor T cell (CAR‐T), cytokine release syndrome (CRS), hypophosphataemia, predictive marker

Key sentences

- Decreased serum iP can be a predictive marker for subsequent severe CRS.
- Excessive renal excretion of iP probably causes post‐CAR‐T hypophosphataemia.

CD19‐targeted chimeric antigen receptor T cell (CAR‐T) therapy is an innovative treatment^1^ for patients with diffuse large B‐cell lymphoma (DLBCL). CAR‐T‐specific complications have yet to be fully controlled; among potential complications, cytokine release syndrome (CRS) is most commonly observed.^2^ It would be extremely useful to be able to predict for each patient, the probability of CRS and its anticipated grade and timing using parameters related to the CAR‐T cell itself or in vivo responses immediately after CAR‐T infusion. Therefore, we performed a retrospective cohort study to identify possible biomarkers using comprehensive screening, to evaluate its accuracy and to reveal relevant mechanisms.

Patients with DLBCL who received tisagenlecleucel (tisa‐cel) or lisocabtagene maraleucel (liso‐cel) from 2018 to 2021 at Kyoto University Hospital were consecutively included in this study. CAR‐T was infused on Day 0, and the general coagulation and chemistry tests were included in biomarker screening. A difference of >10% (either gain or decrease) between Days −1 and 3 was considered significant in primary screening in order to obtain high sensitivity and specificity. In secondary screening, a change of ≥30% before the occurrence of CRS compared with Day −1 was treated as a positive change in order to obtain the higher specificity.

As a result, we enrolled 48 patients treated with tisa‐cel (46 patients) or liso‐cel (two patients; Table 1). In the total cohort, CRS was observed in almost all patients (*N* = 46, 95.8%) at a median (range) of 3 (1–6) days after CAR‐T infusion. In the primary screening (Figure S1), significant changes were observed in serum inorganic phosphate (iP), potassium (K), and magnesium (Mg) levels (all decreased). As a result of the secondary screening, an iP decrease was significantly related to higher incidence of severe CRS (Grade ≥2) with Fisher's exact test (40.0% vs 0.0%, *p* < 0.01; Table S1). Decreased iP after infusion was independent of other patient characteristics and parameters (Table S2), and these observations indicated that serum iP can be used as an early predictive biomarker for subsequent severe CRS.

**TABLE 1:** Patients' characteristics

Characteristic	Value
Total number of patients	48
Age, years, median (range)	59 (20–73)
Sex (female/male), *n* (%)	25 (52.0)/23 (47.9)
Diagnosis, *n* (%)	
Transformed from FL	9 (18.8)
GCB/non‐GCB type	25 (52.1)/23 (47.9)
CD5 positive	11 (22.9)
IPI, *n* (%)	
Low/Intermediate/High	3 (0.63)/24 (50.0)/21 (43.8)
Treatment line, *n* (%)	
4th or later	29 (60.4)
Disease status, *n* (%)	
CR/PR/SD/PD	11(22.9)/14(29.2)/9(18.8)/14(29.2)
CAR‐T product, *n* (%)	
tisa‐cel/liso‐cel	46 (95.8)/2 (4.2)
Occurrence of CRS, *n* (%)	45 (93.8)
Grade 1/2/ 3–4	38 (79.2)/6 (12.5%)/1 (2.1)
Occurrence of ICANS, *n* (%)	3 (6.3)
1/2‐	1 (2.1) / 2 (4.2)
Use of tocilizumab, *n* (%)	29 (60.4)
Use of corticosteroid, *n* (%)	4 (8.3)

Abbreviations: CAR, chimeric antigen receptor; CR, complete remission; CRS, cytokine release syndrome; FL, follicular lymphoma; GCB, germinal centre B‐cell like; ICANS, immune effector cell associated neurotoxicity syndrome.; IPI, International Prognostic Index; liso‐cel, lisocabtagene maraleucel; PD, progressive disease; PR, partial remission; SD, stable disease; tisa‐cel, tisagenlecleucel.

We then investigated trends of iP levels according to clinical courses in relation to CRS. In the iP decrease group (all patients experienced CRS later), serum iP levels were significantly lower as early as Day 1 (median [range] 0.97 [0.42–1.36] mmol/l, *p* = 0.021) and the lowest on Day 4 (median [range] 0.61 [0.36–107] mmol/l, *p* < 0.01), compared to Day 1 (median [range] 1.13 [0.81–1.45] mmol/l). The decreased iP level returned to the normal range after CRS on ~Day 21 (median [range] 1.13 [0.68–1.55] mmol/l, Figure 1). Serum iP level <0.81 mmol/l on Day 3 can also be a risk of consequent occurrence of CRS (data not shown). The whole trends of iP levels including those without iP decrease are shown in Figure S2A. Interestingly, serum Ca level corrected by albumin, which is often coupled with that of iP, was stable in our cohort during the course of CAR‐T therapy (Figure S2B).

**FIGURE 1:**
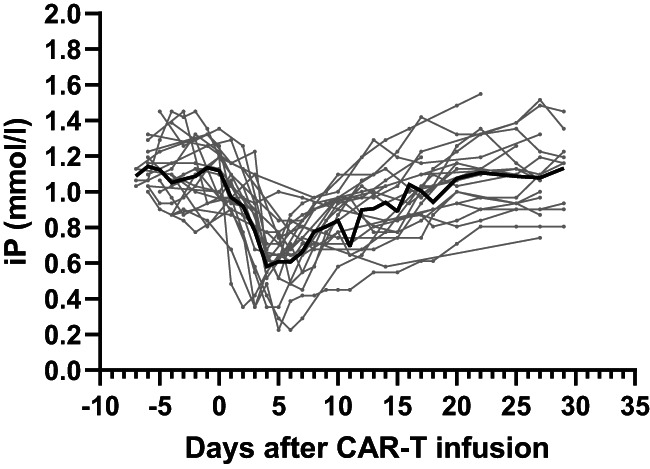
Fluctuations of serum inorganic phosphate (iP) values before and after chimeric antigen receptor T‐cell (CAR‐T) infusion. Chronological trends of iP values are shown in each patient who manifested a significant decrease in iP before CRS. Bold line indicates fluctuation of median values.

Due to the discrepancy in the trends of serum iP and Ca levels after CAR‐T injection, we sought a renal abnormality to explain the significant decrease in serum iP levels. We first checked the urine analyses and found that at the time of the serum iP decrease, renal tubular reabsorption of phosphate (TRP) was as low as 56.2% (median; range 27.2%–71.0%), and the ratio of tubular maximum reabsorption of phosphate to glomerular filtration rate (TmP/GFR) was 5.65 mmol/l (median; range, 3.33–6.62 mmol/l). TRP <80% and TmP/GFR <7.10 mmol/l indicated that the decrease of renal reabsorption and a concomitant increase of renal excretion can explain the iP decrease in serum.

We also checked hormones related to phosphorus dynamics (parathyroid hormone [PTH], activated vitamin D_3_, and fibroblast growth factor 23 [FGF23]) before and after CAR‐T infusions in the most recent 10 cases. PTH levels were comparable between the two time points (Figure S3A), while activated vitamin D_3_ and FGF23 changed significantly (Figure S3B,C). However, increases in activated vitamin D_3_ and decreases in FGF23 generally induce elevations of serum iP levels rather than decreases; therefore, observed decreases in serum iP in post‐CAR‐T cases was independent of these iP‐controlling hormones, or compensation may be insufficient against the significant decrease in iP.

In this retrospective cohort study, we found that: (i) a decrease in serum iP shortly after CAR‐T infusion constitutes a predictive marker for subsequent severe CRS; (ii) fluctuation of serum iP parallels the wax and wane of clinical symptoms of CRS, and (iii) excessive renal excretion of iP appears to be the cause of post‐CAR‐T hypophosphataemia.

This is the first report of the importance of serum iP level monitoring in relation to subsequent CRS. Compared to conventional pre‐CAR‐T phase risk factors for CRS, including systemic tumour volumes,^3^ serum iP is a novel biomarker that reflects the in vivo dynamic response to CAR‐T infusion and can be clinically closely associated with initiation of CRS. A drop in serum iP precedes CRS occurrence by a median of just 1 day, but in clinical settings, this lead time is valuable to prepare physical and human resources for forthcoming severe CRS.^4^ Moreover, the severe hypophosphataemia documented in this study suggests the importance of sufficient supply to prevent muscle weakness, seizures, and heart failure.^5^, ^6^

In order to speculate about the biological mechanism between the serum iP decrease and CRS, we performed comprehensive urine analyses and found that the cause of the serum iP decrease was increased renal excretion due to decreased renal resorption. Hyperinflammatory states such as sepsis and viraemia, induce excessive excretion of iP and hypophosphataemia, due to massive production of serum interleukin 6 (IL‐6) and IL‐6‐induced elevation of FGF23.^7^ Our hormonal analyses revealed that FGF23 was somewhat decreased, and other hormones related to phosphate regulation, including PTH and activated vitamin D_3_, were not the cause of the fluctuation of serum iP after CAR‐T therapy, but followed the drastic changes in iP. Therefore, we speculate that another unknown mechanism must underlie hypophosphataemia before and during CRS after CAR‐T therapy.

In summary, a decrease in serum iP soon after CAR‐T therapy is a useful biomarker to predict occurrence of severe CRS. Monitoring of serum iP levels will be valuable to provide better and safer management for CRS in CAR‐T therapy.

## AUTHOR CONTRIBUTIONS

Naokazu Nakamura and Yasuyuki Arai designed the research, organised the project and performed the statistical analyses. Toshio Kitawaki, Tomoyasu Jo, Chisaki Mizumoto, Junya Kanda, Momoko Nishikori, Kouhei Yamashita, and Akifumi Takaori‐Kondo interpreted the data. All authors critically reviewed the draft and approved the final version of the manuscript.

## FUNDING INFORMATION

The Ministry of Education, Culture, Sports, Science and Technology (MEXT).

## CONFLICT OF INTEREST

The authors declare no competing financial interests.

## Supporting information


Appendix S1

## Data Availability

The data that support the findings of this study are available from the corresponding author upon reasonable request.
