# Supplementary material for: Decreased serum phosphate levels are a useful biomarker to predict occurrence and severity of cytokine release syndrome in chimeric antigen receptor T‐cell therapy
Source: Br J Haematol. 2022 Oct 11;200(1):e1–3. doi: 10.1111/bjh.18504 (PMC10092564; doi:10.1111/bjh.18504)
Supplement: Supplementary file 1 — Appendix S1 [file BJH-200-e1-s001.pdf]

Supplemental Materials

Table S1. Relationship between iP, K, and Mg decreases and CRS

		CRS grade				p value
		0	1	2	3	
iP decrease	N	2	21	0	0	0.001
	Y	0	15	8	2	
K decrease	N	2	34	6	1	0.117
	Y	0	2	2	1	
Mg decrease	N	2	36	8	1	0.083
	Y	0	0	0	1	

Abbreviations: iP, inorganic phosphate; K, potassium; Mg, magnesium; N, no; Y, yes; and CRS, cytokine releasing syndrome.

Table S2. Patient characteristics according to iP values

	Total (N = 48)	IP decrease + (N = 23)	IP decrease - (N = 25)	p value
Median age (range)	59 (20-73)	60 (38-73)	59 (20-71)	0.87
Sex (female/male)	25/23	12/11	13/12	1.00
Diagnosis				
transformed from FL	9 (18.8%)	6 (26.1%)	3 (12.0%)	0.28
GCB type	25 (52.1%)	13 (56.5%)	12 (48.0%)	0.58
non-GCB type	23 (47.9%)	10 (43.5%)	13 (52.0%)	
CD5 positive	11 (22.9%)	5 (21.7%)	7 (28.0%)	0.74
IPI				0.59
Low	3 (0.63%)	2 (8.7%)	1 (4.0%)	
Intermediate	24 (50.0%)	10 (43.5%)	14 (56.0%)	
High	21 (43.8%)	11 (47.8%)	10 (40.0%)	
Treatment lines				
4th or later	29 (60.4%)	13 (56.5%)	16 (64.0%)	0.69
Disease status				0.11
CR	11 (22.9%)	3 (13.0%)	8 (32.0%)	
PR	14 (29.2%)	10 (43.5%)	4 (16.0%)	
SD	9 (18.8%)	5 (21.7%)	4 (16.0%)	
PD	14 (29.2%)	6 (26.1%)	11 (44.0%)	
CAR product				1.00
tisa-cel	46 (95.8%)	22 (95.7%)	24 (96.0%)	
liso-cel	2 (4.2%)	1 (4.3%)	1 (4.0%)	

Abbreviations: IP, inorganic phosphate; DLBCL, diffuse large B cell lymphoma; FL, follicular lymphoma, GCB, germinal center B cell like; FCM, flow cytometry; IPI, standard international prognostic index; CR, complete remission; PR, partial remission; SD, stable disease; PD, progressive disease; tisa-cel, tisagenlecleucel; liso-cel, lisocabtagene maraleucel.

Supplemental Figure Legend

Figure S1. Biomarker screening

Comprehensive biomarker screening was performed using peripheral blood examination data, and % changes on Day3 compared to Day-1 are shown for each parameter. Differences >10% are considered significant in this screening.

Figure S2. Fluctuations of serum iP and Ca values pre- and post-CAR-T infusion

Chronological trends of (**A**) iP and (**B**) Ca values are shown in the whole cohort. Bold line indicates fluctuation of median values.

Figure S3. Changes of iP-related hormones

Difference of iP-related hormones are shown, including (**A**) PTH, (**B**) activated vitamin D3, and (**C**) FGF23. Each dot indicates a value in a patient, and horizontal bars show the median.

Figure S1

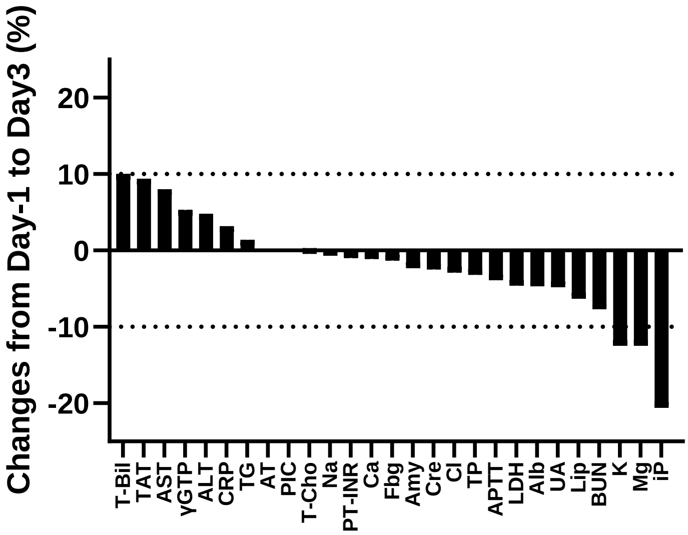

Changes from Day-1 to Day3 (%)
20
10
0
-10
-20
T-Bil
TAT
AST
γGTP
ALT
CRP
TG
AT
PIC
T-Cho
Na
PT-INR
Ca
Fbg
Amy
Cre
Cl
TP
APTT
LDH
Alb
UA
Lip
BUN
K
Mg
iP

Figure S2

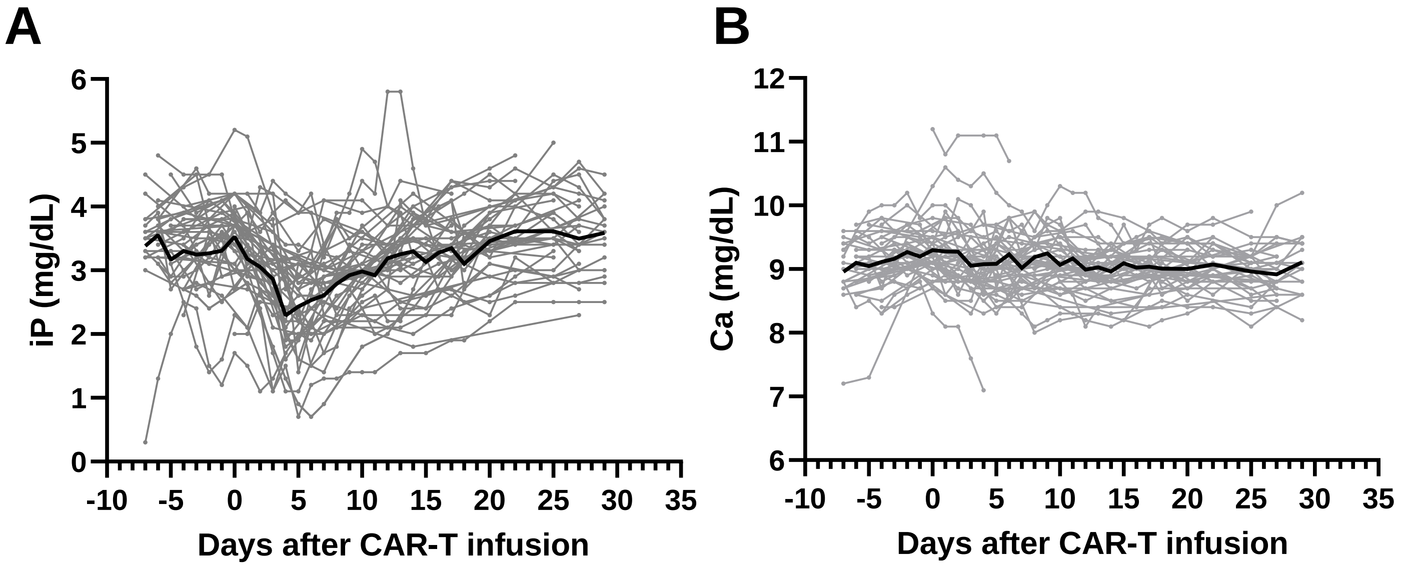
A
iP (mg/dL)
6
5
4
3
2
1
0
-10
-5
0
5
10
15
20
25
30
35
Days after CAR-T infusion
B
Ca (mg/dL)
12
11
10
9
8
7
6
-10
-5
0
5
10
15
20
25
30
35
Days after CAR-T infusion

Figure S3

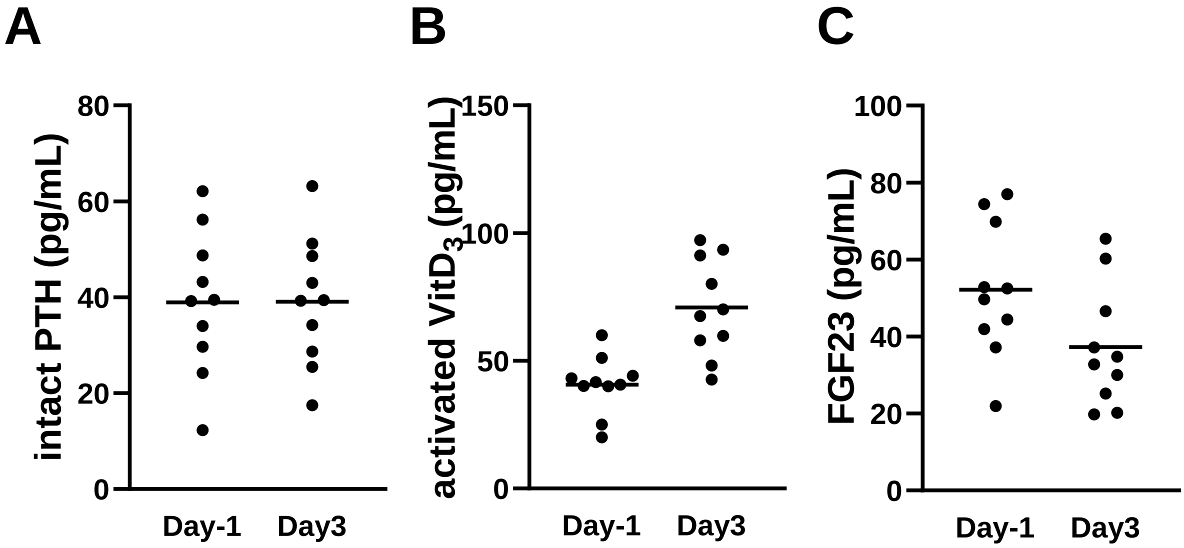
A
B
C
intact PTH (pg/mL)
80
60
40
20
0
Day-1
Day3
activated VitD3 (pg/mL)
150
100
50
0
Day-1
Day3
FGF23 (pg/mL)
100
80
60
40
20
0
Day-1
Day3
